# Factors Associated with the Development of Depression and the Influence of Obesity on Depressive Disorders: A Narrative Review

**DOI:** 10.3390/biomedicines12091994

**Published:** 2024-09-02

**Authors:** Adriano Alberti, David Richer Araujo Coelho, Willians Fernando Vieira, Betine Moehlecke Iser, Rose Meiry Fernandez Lampert, Eliane Traebert, Bruna Becker da Silva, Bruna Hoffmann de Oliveira, Graziela Marques Leão, Gabriela de Souza, Fabiana Meneghetti Dallacosta, Gabriela Kades, Kristian Madeira, Matheus Uba Chupel, Fernando Schorr Grossl, Renan Souza, Ben Hur Soares, Ricelli Endrigo Ruppel da Rocha, Erica da Silva Sipriano, Daniel Fernandes Martins, Lenita Agostinetto

**Affiliations:** 1 Department of Biological and Health Sciences Program in Health Sciences, University of Southern Santa Catarina (UNISUL), Palhoça 88132-260, Brazil; roselampert@mac.com (R.M.F.L.); elisazevedot@gmail.com (E.T.); brunabecker__@hotmail.com (B.B.d.S.); bru.hof.oli@gmail.com (B.H.d.O.); grazielaleao8@gmail.com (G.M.L.); gabrielamicrofisio@gmail.com (G.d.S.); df.martinss@gmail.com (D.F.M.); 2 Graduate Program in Environment and Health, University of Planalto Catarinense—UNIPLAC, Lages 88509-900, Brazil; prof.leagostinetto@uniplaclages.edu.br; 3 Harvard T. H. Chan School of Public Health, Boston, MA 02115, USA; davidcoelho@hsph.harvard.edu; 4 Department of Anatomy, Institute of Biomedical Sciences, University of São Paulo (USP), São Paulo 5508-000, Brazil; williansfvieira@usp.br; 5 Department of Structural and Functional Biology, Institute of Biology, University of Campinas (UNICAMP), Campinas 13083-864, Brazil; 6 Laboratory of Neuroimmune Interface of Pain Research, Faculdade São Leopoldo Mandic, Instituto São Leopoldo Mandic, Campinas 13045-755, Brazil; 7 Department of Biological and Health Sciences Posgraduate Program in Health Sciences, University of Southern Santa Catarina (UNISUL), Tubarão 88704-900, Brazil; betinee@gmail.com; 8 Department of Biosciences and Health, University of West Santa Catarina, Joaçaba 89600-000, Brazil; fabiana.dallacosta@unoesc.edu.br (F.M.D.); kadesgabriela042@gmail.com (G.K.); fernando_grossl@hotmail.com (F.S.G.); renan-souza@unoesc.edu.br (R.S.); 9 Department of Mathematics and Health Sciences, University of the Extreme South of Santa Catarina (UNESC), Criciúma 88806-000, Brazil; kristian@unesc.net (K.M.); ericassipriano@gmail.com (E.d.S.S.); 10 Hurvitz Brain Sciences, Biological Sciences Platform, Sunnybrook Research Institute, Toronto, ON M4N 3M5, Canada; matheus.chupel@utoronto.ca; 11 Department of Physical Education and Physiotherapy, University of Passo Fundo, Passo Fundo 99052-900, Brazil; benhur.upf@gmail.com; 12 Department of the Graduate Program in Development and Society—PPGEDS (UNIARP), University of Alto Vale do Rio do Peixe, Caçador 89500-199, Brazil; ricellie@uniarp.edu.br

**Keywords:** obesity, depression, mental health, inflammation

## Abstract

Depression affects several aspects of life, including socioeconomic status, relationships, behavior, emotions, and overall health. The etiology of depression is complex and influenced by various factors, with obesity emerging as a significant contributor. This narrative review aims to investigate the factors associated with the development of depression, with a particular focus on the role of obesity. The literature search was conducted on PubMed, Embase, and PsycINFO from May to July 2024. The review highlights the impact of environmental and socioeconomic conditions; lifestyle choices, including physical activity and dietary habits; stress; traumatic experiences; neurotransmitter imbalances; medical and psychological conditions; hormone fluctuations; and epigenetic factors on depression. A key emphasis is placed on the inflammatory processes linked to obesity, which may drive the bidirectional relationship between obesity and depression. The findings suggest that obesity is associated with an increased risk of depression, potentially due to chronic inflammation, neurochemical dysregulation, and the emotional and social challenges related to weight stigma and obesity management. Understanding these interconnected factors is important for developing targeted interventions to address both obesity and depression, leading to improved quality of life for those affected.

## 1. Introduction

The origins of depression can be traced back to studies from ancient Greece, where Hippocrates, in the 5th century BC, defined this disorder as melancholia, associating it with changes that resulted in profound sadness, insomnia, lack of appetite, and a desire for death [1,2]. The modern concept of depression originated from earlier diagnostic formulations of melancholia over a century-long period, from the 1780s to the 1880s. This historical outline traces the evolution of these ideas through the writings of 12 authors, highlighting the central roles played by the concepts of educational psychology and comprehensibility. Five of these authors, who wrote between 1780 and 1830, such as Cullen, Pinel, and Esquirol, defined melancholia as a disorder of the intellect or judgment, a form of “partial insanity” often associated with sadness, though not always [3]. 

Mental disorders are neuropsychiatric conditions that result in psychological or behavioral changes, affecting daily activities and limiting social interaction [4]. These conditions represent a barrier to an individual’s engagement with their environment, restricting their freedom and social interaction [5]. 

Globally, mental illnesses have received significant attention due to the increasing incidence in both developed and developing countries. It is estimated that around 700 million people worldwide experience mental and neurological disorders [6]. Additionally, one in four people will face these conditions at some point in their lives, making it uncommon for families not to have a member affected by mental disorders [7]. Depression is one of the most prevalent mental disorders and is the leading cause of disability worldwide. Its social and economic impacts are significant, with depression and anxiety estimated to result in an annual productivity loss exceeding one trillion dollars [8]. Despite this, investment in the treatment of mental disorders remains disproportionate to societal needs, especially in low- and middle-income countries, where investment in mental health accounts for less than 1% of the total health budget, and only 20 to 40% of people receive adequate treatment. Early intervention is crucial; however, individuals with depression often hide symptoms due to stigma and shame, hesitating to seek help from psychiatrists, especially in the early stages [8]. 

The causes of depression cannot be attributed to a single factor but rather to an interaction of various factors related to the emergence of depressive symptoms, which can be social, psychological, and biological, potentially relating to both recent and early adverse life events [9]. Depression is associated with an inflammatory state. The activation of inflammatory pathways in the brain is a significant producer of excitotoxicity and an inducer of oxidative stress, contributing to neuronal damage in patients with mental disorders. In major depression, there is an increase in pro-inflammatory hormones and cytokines in the plasma, along with indicators of oxidative stress. These changes result in alterations in cognitive and executive functions due to brain inflammation. Inflammatory biomarkers have emerged as crucial tools for diagnosing, classifying the severity, and guiding the treatment of major depression [10]. 

Like depression, obesity is a disease associated with an inflammatory condition. It has been established that both obesity and depression induce inflammatory states in the body, and inflammation may mediate the bidirectional relationship between these two disorders. Preclinical studies consistently show that obesity is associated with an increased risk of developing depression [11]. This article offers a new perspective by integrating recent evidence on the connection between obesity and depression, areas that are constantly evolving due to new scientific discoveries. What sets this study apart from others is the emphasis on the bidirectional interactions between obesity and depression, particularly how obesity can be both a risk factor and a consequence of depression. Additionally, the article addresses the therapeutic and public health policy implications, suggesting that prevention and treatment strategies should consider this complex interrelationship. By providing an updated and integrated review, this article contributes to a better understanding of the most effective strategies to address these public health challenges.

Given the significant burden posed by depression and its complex relationship with obesity, this article aims to analyze the factors associated with the development of depression, with a particular focus on the influence of obesity. This review seeks to synthesize existing knowledge, identify research gaps, and propose new directions for future investigations.

Depression and obesity are widespread conditions with significant public health implications and economic impact. Obesity and depression present an increased risk of developing secondary conditions such as cardiovascular diseases, diabetes, fibromyalgia, reduced quality of life, and sedentarism. The association between depression and obesity is well documented and has been examined extensively [12].

Depression and obesity are common conditions with a significant public health burden and often coexist among the same individuals. This relationship is bidirectional, with the presence of one condition increasing the risk of developing the other [11,12]. This narrative review will discuss the relationship between these factors and investigate the causes of depression [9,10]. A search on PubMed, Embase, and PsycINFO was conducted from May to July 2024 focusing on recent studies exploring the development of depression and its relation with obesity. 

## 2. Depressive Disorders

Epigenetic factors introduce a new perspective into therapeutic approaches, as most diseases are not caused by a single gene and are significantly influenced by the environment. Epigenetic inheritance is implicated in various mental disorders, including depression. In summary, these epigenetic modifications can be categorized into three main types: DNA methylation, histone modification, and regulation of non-coding RNA (ncRNA). This review identified several genes associated with depressive disorders using bioinformatics tools and gene databases, including SLC6A4, COMT, TPH2, FKBP5, MDD1, HTR2A, and MDD2 [9]. 

Depression, in turn, has various causes ranging from genetic and epigenetic factors to environmental conditions, stress, and nutritional deficiencies. Studies show that both genetic and environmental factors play an important role in the development and manifestation of depression. Additionally, low levels of hormones such as dopamine and serotonin are also significant factors. Furthermore, there are clinical and molecular characteristics associated with depression that cannot be fully explained by traditional genetic and environmental factors alone [9].

The main mechanisms for chemical imbalances in the brain involve blocking the reuptake of neurotransmitters involved (serotonin, noradrenaline, and dopamine), thereby increasing their concentration in the synaptic terminal; and inhibiting enzymes that metabolize neurotransmitters, allowing them to continue acting [11].

## 3. Factors Associated with the Development of Depression

### 3.1. Environmental Factors 

Environmental conditions play an important role in the onset of depression, with evidence suggesting that the connection between the cerebellum and depression may be influenced by these factors. It can then be postulated that some environmental factors alter cerebellar functionality and lead to vulnerability to depression [13]. 

Among the factors that may be linked to depression is chronic stress: prolonged exposure to stress, whether due to work problems, financial difficulties, or turbulent relationships, can significantly increase the risk of depression. The release of cytokines resulting from chronic stress can lead to the differentiation of peripheral CD4+ cells into various phenotypes. Among these, Th17 cells have attracted much attention due to their high pathogenic potential in central nervous system (CNS) diseases [14].

Trauma and abuse situations are also factors that may be linked to the development of depression, as traumatic experiences, such as physical, emotional, or sexual abuse, especially during childhood, are strongly associated with the development of the disorder [15]. Social isolation is also among some of the factors, as the lack of social support, loneliness, and isolation can contribute to feelings of sadness and hopelessness, increasing the risk of depression. A study by Ge et al. [16] highlights that social isolation indicators accounted for 2.4% of the variation in depressive symptoms, while loneliness explained a higher percentage, 9.7%, among various indicators of social isolation and loneliness in adults aged 21 and over living in the community.

Furthermore, environmental conditions like lack of sunlight can affect mood and contribute to depression. Insufficient sunlight exposure, especially in colder and darker climates, can lead to seasonal affective disorder (SAD), a form of depression typically occurring during winter months. According to Lin et al. [17], spending an average of 1.5 h per day under outdoor light is associated with a lower risk of depression, regardless of genetic predisposition. Moderate outdoor light exposure can reduce the risk of depression, even in those with a higher genetic risk.

The prevailing theory suggests that the lack of sunlight may interfere with the proper functioning of the hypothalamus, affecting melatonin production—a hormone involved in sleep regulation that can be excessively produced in SAD. Additionally, reduced sunlight exposure can lower levels of serotonin, a hormone that influences mood, appetite, and sleep, and whose deficiency is linked to depression. Sunlight also regulates the circadian rhythm, and reduced exposure during winter can disrupt this biological clock, leading to SAD symptoms. Genetic factors can also heighten susceptibility to SAD, as the condition can occur in some families [18].

Environmental factors related to xenobiotics, such as agricultural pesticides, are also associated with depression. Both rural workers engaged in conventional agriculture and individuals directly or indirectly exposed to pesticides are at risk of contamination, with pesticides considered a significant public health issue in Brazil and globally [19,20,21,22,23,24]. The risks linked to these chemicals include chronic diseases and psychiatric disorders, including depression, anxiety, and even suicide in extreme cases [25,26]. In Brazil, studies have shown a notable statistical association between farmers who experienced depression and cases of pesticide poisoning [20].

Among the primary pesticide classes affecting the nervous system are insecticides, particularly organophosphates and pyrethroids [26]. These substances are widely used in agricultural regions and are commonly associated with poisoning cases [20,27,28,29]. Organophosphate insecticides inhibit acetylcholinesterase (the enzyme responsible for breaking down acetylcholine—Ach—in the synaptic cleft), leading to changes in the central nervous system. The resultant accumulation of Ach causes hyperstimulation of Ach receptors on postsynaptic neurons, which can lead to serotonergic alterations associated with mental disorders such as depression, anxiety, and aggressive behavior [26]. Scientific evidence indicates that adolescents living near agricultural areas exhibit a 0.96 unit increase in depressive symptoms for every 10% reduction in AchE activity, with stronger associations observed in girls and older adolescents compared to boys and younger ones [21].

Additionally, pyrethroid insecticides are linked to neurotoxicity, which may result in delayed reflexes, reduced cognitive ability, and alterations in neurotransmission, potentially leading to respiratory depression in severe cases [26]. Exposure to these pesticides disrupts dopaminergic, serotonergic, and neurological functions [30]. Research has found a positive association between environmental exposure to pyrethroids and diagnoses of depression and anxiety, suggesting that such exposure may be linked to the late onset of depression throughout life.

### 3.2. Changes in Neurotransmitters

Changes in neurotransmitters are believed to play a significant role in the development of psychiatric disorders. In depression, there is a decrease in the number of neurotransmitters released, but the reuptake pump and enzyme continue to function normally. Thus, a receptor neuron captures fewer neurotransmitters, and the nervous system operates with fewer neurotransmitters than is normally needed [31]. There is a relationship between the three main monoamine neurotransmitters in the brain (dopamine, norepinephrine, and serotonin) and the specific symptoms of MDD. The increase or decrease in these neurotransmitters is linked to specific symptoms, highlighting that specific neurochemical mechanisms are responsible for certain symptoms of depression. Thus, antidepressants can be targeted at specific neurotransmitters to treat these symptoms [32].

Serotonin is widely recognized for its influence on mood, sleep, and appetite [33]. Low levels of serotonin are frequently associated with depression, potentially leading to symptoms such as persistent sadness, irritability, changes in sleep patterns, and loss of interest in previously enjoyable activities. Many antidepressants, such as selective serotonin reuptake inhibitors (SSRIs), work by increasing serotonin levels in the brain to alleviate these symptoms [34,35].

Norepinephrine plays a crucial role in regulating attention, stress response, and alertness. Alterations in norepinephrine levels are associated with symptoms of depression, including lack of energy, decreased interest in activities, and difficulties concentrating. Tricyclic antidepressants and some serotonin–norepinephrine reuptake inhibitors (SNRIs) work by increasing norepinephrine levels, which help alleviate these depressive symptoms [36].

Dopamine is a fundamental neurotransmitter involved in pleasure, reward, and motivation mechanisms [37]. Insufficient levels of dopamine are associated with anhedonia (the inability to feel pleasure), lack of motivation, and loss of interest, symptoms often observed in depression. Regulating dopamine levels is a therapeutic goal in some antidepressants and treatments for mood disorders, aiming to restore dopaminergic balance and improve patients’ emotional well-being [38].

In addition to the main neurotransmitters, other substances such as gamma-aminobutyric acid (GABA) and glutamate also play significant roles in depression [39]. GABA is an inhibitory neurotransmitter that decreases neuronal excitability, promoting a calming effect on the brain. On the other hand, glutamate is an excitatory neurotransmitter that increases neuronal activity. Imbalances between GABA and glutamate can compromise emotional stability and the ability to respond to stress. These imbalances are implicated in the pathophysiology of depression, suggesting that modulating these neurotransmitters may be a potential therapeutic strategy to improve depressive symptoms [39,40].

### 3.3. Prolonged Stress Situations and Traumatic Experiences

Since the beginning of humanity, people have suffered from stress. Stress is a well-known factor significantly involved in the onset of nearly all major depressive disorders [41] and it is generated by cumulative stressful situations. A stressful situation, whether environmental or psychological, can trigger a series of hormonal reactions resulting in physiological changes. The activation of the sympathetic nervous system in this way provokes an acute stress response known as the fight-or-flight response. This response allows the individual to either confront the threat or escape the situation. The release of adrenaline and noradrenaline by the adrenal medulla causes widespread activation of the sympathetic system throughout the body. The physiological changes resulting from this activation include increased blood pressure, greater blood flow to active muscles, reduced blood flow to organs not essential for rapid motor activity, increased blood clotting rate, elevated cellular metabolism rates throughout the body, increased muscle strength, heightened mental activity, elevated blood glucose concentration, and increased glycolysis in the liver and muscles. The cumulative effect of these changes enables the person to perform more intense activities than usual. Once the perceived threat dissipates, the body returns to baseline levels [42].

There are various types of traumatic stimuli, including catastrophic events such as wars, natural disasters like earthquakes, and personal traumas resulting from neglect and physical, psychological, or sexual abuse. Traumatic events can be classified as type I or type II traumas, and their impacts on individuals vary not only by the severity and duration of the traumas but also by how the individuals themselves evaluate these events. Individual reactions to trauma can include post-traumatic stress disorder (PTSD), complex PTSD, and trauma-related depression [43].

Prolonged stress situations and traumatic experiences can have devastating and long-lasting effects on individuals’ mental and physical health. Chronic stress is associated with a range of health problems, including cardiovascular diseases, hypertension, diabetes, and immune system dysfunctions [44]. Furthermore, continuous stress can negatively impact the brain, resulting in structural and functional changes, such as the reduction of hippocampal volume, a region crucial for memory and emotional regulation. These physiological changes can exacerbate the symptoms of mental disorders, creating a vicious cycle of deteriorating mental and physical health [45].

Adverse psychosocial exposures early in life have increasingly been associated with significant physical and emotional symptoms. These include obesity [46,47], depression [48], attention-deficit/hyperactivity disorder (ADHD) [46], and respiratory diseases, with symptom severity correlating with the number of adversities experienced [46]. Adverse childhood experiences (ACEs) demonstrate an observable and lasting impact on the functional dynamics of the brain–body–mind, even among individuals who experience trauma without developing post-traumatic stress disorder (PTSD) [47].

ACEs can result in persistent neurobiological changes, increasing the risk of developing mental disorders later in life [49]. The systematic study of ACEs garnered scientific interest in the 1990s following the release of results from the ACE study [50,51]. Conducted in San Diego by the Kaiser Department of Preventive Medicine in collaboration with the United States Centers for Disease Control and Prevention (CDC), this study is recognized as a milestone in investigating childhood abuse and neglect as risk factors associated with adverse health and well-being outcomes throughout life [50]. Since then, numerous epidemiological studies have emphasized growing global concern about the short- and long-term consequences of child maltreatment [52,53,54].

It has been demonstrated that childhood adversities contribute to inflammation in both children and adults [55,56]. ACEs manifest with a systemic inflammatory response, increasing the risk of developing obesity as a comorbidity. Elevated inflammatory response to stress can also increase the desire for tasty foods and impair bodily function [47]. Systemic inflammation, largely stemming from these adversities, is associated with mental health problems, including depression [54] and obesity [52,53]. Individuals who have experienced ACEs are up to 46% more likely to develop obesity in adulthood [47]. The timing (e.g., developmental stage) and duration (e.g., episodic versus continuous) of ACEs may also have significant implications for their effects [57].

Chronic inflammation is recognized as one of the main mechanisms through which ACEs influence the development of long-term diseases such as cardiovascular diseases and depression [58]. There is an association between ACEs, depression, and elevated levels of interleukin-6 (IL-6), as well as an association between high levels of C-reactive protein in depressed elderly individuals and ACEs in childhood [59]. Studies based on data from 8,810 participants in a British birth cohort indicate that exposure to parental offense, physical abuse, and emotional neglect is strongly associated with elevated inflammatory levels in mid-life [55].

Additional research shows that parental separation/divorce can result in higher levels of IL-6 compared to other types of ACEs, especially in 9-year-old children [56]. In large-scale studies, a class of “High Adversity” (6%) has been identified, characterized by multiple adverse exposures such as physical and emotional abuse, witnessing domestic violence, and living with adults with substance abuse problems. This class, along with the “Child Abuse” class (16%), showed a higher prevalence of MDD compared to the “Low Adversity” class (69%) [54].

Detailed analysis using data from the National Longitudinal Study of Adolescent to Adult Health in the US revealed that the Child Abuse class showed a significantly increased risk of depressive symptoms, negatively mediated by self-esteem [48]. Another large-scale study involving 105,759 anonymously surveyed adolescents confirmed a strong association between ACEs and obesity. Factors such as male sex, older age, poverty conditions, and residence in non-metropolitan areas were significantly correlated with higher BMI among adolescents exposed to ACEs [52].

Furthermore, a detailed analysis of data from 6,942 adolescents between 9 and 13 years old in Ireland showed that the interaction between ACEs and low income independently predicts the risk of obesity in early adolescence. These findings underscore the lasting impact of ACEs on the development of conditions such as overweight and obesity during adolescence [53].

Sexual abuse appears to have a particularly significant impact on childhood obesity compared to other ACEs. Additionally, the co-occurrence of multiple ACEs may be associated with a higher risk of childhood obesity, with the effect of these adversities on obesity development manifesting over 2 to 5 years following the adversities [57]. ACEs can interfere with psychosocial aspects and neuroendocrine development, contributing to obesity due to associated impairments in self-regulation, appetite, and psychopathology [57].

Finally, evidence from a study involving 1,335 families in the United States indicated that exposure to ACEs in early childhood was associated with a variety of health problems, including obesity, respiratory issues, and attention difficulties, reflecting an increased risk of compromised health by 11 years of age [46].

During certain stages of development, trauma can cause significant neurobiological changes, such as volumetric and functional variations in the amygdala and hippocampus [60]. Biological manifestations of childhood abuse include increased inflammation, heightened hypothalamic–pituitary–adrenal (HPA) axis reactivity, sleep disturbances, and immune system suppression [47]. These contribute to dysregulated stress responses, abnormal cortisol levels, compromised immune function, and increased inflammatory markers [61], such as elevated C-reactive protein, as well as an amplified amygdala response to emotionally negative stimuli, along with reduced gray matter volume in the hippocampus [49]. Decreased hippocampal region activity contributes to depression, while a structural anomaly in the superior insula region activates the brain to receive more negative experiences. These findings are consistently observed in individuals with and without psychiatric diagnoses, underscoring the enduring effects of adverse experiences on mental and physical health throughout life [49].

### 3.4. Medical Conditions

The presence of adverse emotional conditions, such as depression, can trigger or exacerbate chronic health problems. These emotional changes are intrinsically related to eating behaviors, influencing individuals to seek emotional relief and gratification through food consumption to address possible affective deficiencies. An individual’s eating behavior encompasses a range of actions related to food, from choice to consumption. This behavior can be influenced by the interaction of biological, psychological, and social factors in which the individual is embedded. Any alteration in these aspects will directly influence their eating behavior [62]. 

Changes in eating habits are common in individuals with MDD. In one study, 46% of participants diagnosed with MDD exhibited atypical features and reported increased appetite. Among depressed patients without atypical features, 18% reported increased appetite, while 50% reported decreased appetite [63]. These eating habits directly impact quality of life and social interaction, as well as increase the risk of non-communicable chronic diseases such as obesity, diabetes, and hypertension [64]. 

Research demonstrates a significant association between depression and non-communicable chronic diseases. A meta-analysis published by DeGroot et al. [65] showed a significant and consistent association between depressive symptoms and diabetes complications. In the Brazilian population, the findings are similar: a cross-sectional study aimed at evaluating the presence of depressive symptoms in patients with diabetes treated at the Ciências Médicas Outpatient Clinic concluded that patients with diabetes had a considerable prevalence of depression, with a prevalence of 37.5% in the analyzed sample [66]. Additionally, Adamis et al. observed that a depressive state is associated with elevated blood pressure levels, showing a strong correlation between these pathologies [67]. In patients with arterial diseases, depression increases the risk of acute myocardial infarction by 1.5–4.5 times [68]. Authors highlight the higher prevalence of angina in patients with or without coronary artery disease [69].

The interrelationship between chronic pain and depression is complex, suggesting a symptomatic link between both conditions. Individuals suffering from chronic pain often experience depressive symptoms, attributed to neurotransmitters such as glutamate, serotonin, and GABA that are shared between these pathologies [70]. The nature and intensity of chronic pain, along with the degree of depression, exert a direct influence on this relationship. On the other hand, the association between obesity and these conditions also requires further investigation. However, some studies already point to a connection between obesity, high depression scores, and the presence of chronic pain [71]. Additionally, evidence suggests that the pharmacological management of chronic pain and depression can negatively impact body weight, highlighting the importance of a careful approach in prescribing medications and the need for innovative treatment strategies [72].

Another complex interrelation between obesity, depression, and Alzheimer’s disease (AD) is extremely relevant in contemporary health [73,74]. Not only may early depression increase the risk of developing AD, but also late-life depression can anticipate its symptoms [75]. This phenomenon is partly driven by decreased synaptic framework and neuroplasticity, marked by reduced brain-derived neurotrophic factor (BDNF) and affected by neuroinflammatory processes at central and peripheral levels common to these conditions [74,75,76]. It is estimated that about 23% of obese individuals in the United States also suffer from depression, and this depressive condition, in turn, increases the risk of obesity by 37% [75]. Additionally, obesity in mid-life is correlated with a higher risk of AD, whereas in older ages, obesity, especially when metabolically healthy, may offer some protection against this pathology [75]. Therefore, the development of therapeutic approaches should be based on lifestyle changes, as these are multifactorial and complex conditions, with several other potentially modifiable risk factors including hypertension, dyslipidemia, smoking, and sedentary behavior [73,74].

Adverse emotional conditions, such as depression, can trigger and worsen chronic health issues. Emotional distress often leads individuals to engage in unhealthy eating behaviors, such as consuming high-calorie foods to cope with their feelings. This can result in a cycle of poor health habits, reduced physical activity, and overall diminished quality of life, which increases the risk of chronic non-communicable diseases like obesity, diabetes, and hypertension. Studies have consistently linked depression with these conditions. For example, DeGroot et al. [65] found a strong association between depressive symptoms and complications in diabetes. Research in Brazil also shows high rates of depression among type 2 diabetes patients, with a correlation between depressive symptoms and diabetic neuropathic complications. Adamis et al. [67] highlighted the relationship between depression and elevated blood pressure, and additional evidence shows that depression increases the risk of acute myocardial infarction in patients with arterial diseases. These findings underscore the importance of addressing these interconnected health issues through integrated treatment and prevention strategies.

### 3.5. Changes in Hormone Levels

Changes in hormone levels are a significant factor in the development of depression, reflecting the intricate interplay between the endocrine system and the nervous system. Hormones and neurotransmitters share common pathways and receptor sites in areas of the brain linked to mood, particularly through the hypothalamic–pituitary–gonadal axis [77]. Disruptions in this axis can contribute to the onset of depression. For example, the HPA axis, which responds to stress, has been extensively studied for its role in the pathophysiology of anxiety and depression, and its influence on cognitive functioning [78].

Stress hormones are crucial in the body′s response to both physical and emotional stress. When exposed to stress, the brain activates the sympathetic nervous system, which triggers the release of cortisol and adrenaline [60]. Cortisol, known as the primary stress hormone, regulates metabolism, elevates blood sugar levels, and suppresses non-essential functions such as the immune and digestive systems to provide rapid energy [79]. Adrenaline increases heart rate, dilates blood vessels, and improves respiratory function, preparing the body for physical action [60]. In cases of depression, there is often an imbalance in the regulation of these stress hormones, with elevated cortisol levels persisting even in the absence of stress, which can exacerbate symptoms. Furthermore, chronic stress has been shown to negatively impact brain regions involved in mood regulation and to increase systemic inflammation, both of which are associated with the development and progression of depression [80].

Natural hormonal fluctuations, such as those occurring during pregnancy and menopause in women, and andropause in men, can also significantly affect mental health [81,82]. These hormonal changes contribute to the complexity of depression, highlighting the need for a nuanced understanding of how endocrine disruptions intersect with psychological well-being.

### 3.6. Substance Abuse

Substance use involves the consumption of various substances, including alcohol, tobacco products, drugs, inhalants, and other substances that can be ingested, inhaled, injected, or otherwise absorbed by the body. This usage can potentially lead to dependence and other adverse effects [83]. When dependence develops, it results in substance abuse, which has significant negative repercussions for the individual. This behavior can compromise both physical and mental health and negatively impact social, professional, and family life. Substance abuse includes both the excessive and improper use of legal substances (such as alcohol, tobacco, and prescription medications) and illegal drugs (such as cocaine, heroin, methamphetamine, and marijuana) [84].

The interplay between depression and substance use is intricate and bidirectional. Individuals experiencing depression may turn to alcohol or drugs as a form of self-medication to temporarily alleviate their emotional symptoms. However, the chronic use of these substances often exacerbates depression, perpetuating a vicious cycle. Additionally, substance use can induce depression through neurochemical alterations in the brain, the negative consequences associated with abuse, and withdrawal symptoms [84,85,86].

Substance abuse, including the misuse of alcohol and drugs, has a profound effect on depression. Chronic substance use alters brain chemistry, particularly affecting neurotransmitters such as serotonin and dopamine, which are essential for mood regulation [87]. These alterations can impair the brain’s normal functioning, thereby intensifying depressive symptoms. Furthermore, the cycle of substance dependence and withdrawal often leads to severe mood swings, exacerbating emotional instability and obstructing recovery [87,88].

### 3.7. Socioeconomic Factors

Socioeconomic conditions play a crucial role in the prevalence and severity of depression and other mental disorders. Research indicates that individuals from poorer socioeconomic backgrounds have fewer opportunities and resources, which can significantly influence the occurrence of depressive episodes [89]. Several studies have demonstrated a higher prevalence of depression among individuals from less advantaged social classes [90,91].

A study conducted in 2018 revealed that depressive symptoms were notably associated with lower levels of education and belonging to economic classes D or E [89]. Additionally, a 2023 review emphasizes that depression is significantly influenced by various social and economic factors, including low wages, lack of financial reserves, and homeownership status [91].

Financial difficulties impact mental health across short-, medium-, and long-term periods. Consistent evidence shows a positive association between financial stress and depression, as highlighted by a systematic review encompassing 40 observational studies [92]. This relationship is evident in both high-income and low- to middle-income countries, though stronger effects are observed among individuals with lower income or wealth. In addition to “social causation,“ mechanisms such as psychological stress and social selection contribute to the impact of financial stress on depression [92,93].

### 3.8. Psychological Disorders 

Depression is frequently linked with a range of other mental disorders, creating a complex and challenging clinical picture [94]. This association is particularly strong with generalized anxiety disorder (GAD), sharing not only overlapping symptoms such as nervousness and concentration difficulties but also significant clinical comorbidity. Individuals with depression often exhibit chronic anxiety symptoms, while those with GAD may show depressive symptoms, including anhedonia and profound dysphoria. Both disorders appear to have similar neurobiological underpinnings, influenced by neurochemical imbalances as well as genetic and environmental factors, making diagnosis and treatment particularly complex [95,96].

The relationship between depression and bipolar disorder is intricate, involving a complex interplay between opposing mood states. Bipolar disorder is characterized by alternating episodes of mania—marked by intense euphoria and impulsivity—and depressive episodes that share symptoms with major depressive disorder. Research underscores that many patients with bipolar disorder experience significant depressive episodes, though the critical distinction lies in the presence of distinct manic or hypomanic episodes, which differentiates bipolar disorder from unipolar depression [97].

During depressive episodes of bipolar disorder, symptoms include a persistently depressed mood, alterations in sleep and appetite, feelings of hopelessness, and low self-esteem. Effective management typically involves mood stabilizers to address both depressive and manic episodes due to the cyclical and unpredictable nature of the disorder. Understanding this dynamic is crucial for managing symptoms and improving the quality of life for patients [98,99].

The interplay between depression and borderline personality disorder (BPD) is also complex and pervasive. BPD is characterized by chronic emotional instability, impulsivity, turbulent interpersonal relationships, and an unstable self-image. Individuals with BPD frequently experience intense and recurrent depressive episodes, often alongside mood disorders such as MDD and dysthymia. These depressive symptoms can exacerbate the emotional intensity and impulsivity inherent to BPD [100,101].

The intersection of depression and BPD is evident in the high incidence of suicide attempts among patients with BPD, along with self-injurious behaviors, which are often linked to impulsivity and hopelessness exacerbated by depression. The overlap in emotional and behavioral symptoms between these disorders presents significant challenges for differential diagnosis and the development of effective therapeutic interventions [102,103].

The relationship between panic disorder and depression is well documented. Panic disorder is characterized by recurrent and unexpected panic attacks, typically accompanied by intense physical symptoms such as palpitations, shortness of breath, tremors, and sweating. Many individuals with panic disorder also develop depressive disorders, either due to the debilitating nature of persistent panic attacks or shared biological and psychosocial factors [104,105].

The association between obsessive–compulsive disorder (OCD) and depression is also well established. Individuals with OCD often experience episodes of MDD, with both disorders sharing symptoms such as anxiety, hopelessness, and low self-esteem, which complicate the clinical presentation and treatment. Patients with OCD may face additional challenges due to the interference of obsessive and compulsive symptoms with daily functioning, while depression can exacerbate these difficulties. Effective treatment generally requires an integrated approach that addresses both OCD and depression, incorporating antidepressants, psychotherapy, and other interventions tailored to both conditions [106,107].

The relationship between ADHD and depression is multifaceted. Individuals diagnosed with ADHD are at an elevated risk of developing depressive symptoms throughout their lives. Research indicates that a significant proportion of people with ADHD experience depression, driven by factors such as chronic emotional regulation difficulties, low self-esteem linked to academic and interpersonal challenges, and the complexities of managing ADHD symptoms. Additionally, neurochemical imbalances and alterations in executive brain functions, which are characteristic of ADHD, may contribute to the increased susceptibility to depression [108,109,110].

### 3.9. Food Preferences

Individuals with depression often have a preference for fast food, snacks, and foods of low nutritional quality, characterized by high energy content. Those experiencing severe depression tend to follow an unhealthy diet, marked by reduced consumption of fruits and vegetables (FV), fish, chicken, milk, and grains [111,112]. Meta-analyses have shown that higher FV consumption is inversely related to depressive symptoms, indicating a 14% lower risk of depressive symptoms in individuals with higher FV intake. Each 100 g increase in daily FV consumption is associated with a 5% reduction in the prevalence of depression [113]. This association may be attributed to the minerals, vitamins, amino acids, phytochemicals, and antioxidant compounds found in FV, which positively influence depression [114,115].

A meta-analysis of longitudinal studies revealed that adherence to a Mediterranean diet—characterized by high consumption of fruits, vegetables, legumes, grains, fish, and olive oil, and low consumption of meat and dairy products, with moderate alcohol intake—was associated with a lower risk of depression (risk ratio (RR), 0.67) compared to those with less adherence [116]. Conversely, a recent meta-analysis found that the consumption of snacks, sugary drinks, and ready meals was linked to an increased risk of depression (RR, 1.28); every 10% increase in the intake of these foods per daily calorie consumption was associated with an 11% increased risk of depression among adults [117]. 

In a study conducted in Finland, a diet rich in vegetables, fruits, chicken, fish, whole grains, legumes, berries, and low-fat cheese was compared to a Western diet consisting of processed foods such as sausages, French fries, fast food, and sweets like ice cream and chocolates. Among middle-aged Finnish men, a higher adherence to the healthy dietary pattern showed a 25% reduction in the risk of depressive symptoms. In contrast, adherence to the Western diet was associated with a 41% increase in depressive symptoms [118].

A recent review of 21 studies from various countries identified a dietary pattern characterized by high consumption of fruits, vegetables, whole grains, fish, olive oil, low-fat dairy, antioxidants, and low intake of animal foods as being associated with a reduced risk of depression [119]. A study conducted in Korea that evaluated middle-aged individuals (40 to 60 years old) found that poor dietary quality, inadequate protein intake, and irregular meals were associated with an increased risk of depression [120].

The literature highlights the significance of meal timing for both physical and mental health. Wilson et al. (2020) identified three meal timing patterns: grazing (distributed consumption throughout the day), traditional (higher consumption centered around breakfast, lunch, and dinner), and late (skipped or delayed breakfast with higher nighttime consumption). The late meal pattern was associated with an increased risk of mood disorders, while the traditional pattern was linked to a substantially lower risk of mood disorders. This suggests that non-traditional eating habits, such as skipping or delaying breakfast, may be related to mood disorders [121].

The consumption of ultra-processed foods, often used as substitutes for lunch or dinner, is strongly associated with an increased risk of depression [122]. Furthermore, metabolic disorders like obesity are closely related to depression and the consumption of fast food, suggesting a shared biological mechanism [123,124].

Foods with a high glycemic index, such as soft drinks and sweets, are associated with inflammatory markers and oxidative stress, which in turn are linked to chronic non-communicable diseases and depressive symptoms [125,126,127,128]. A study by Knüppel et al. (2017) identified an increased risk of depressive symptoms related to high intake of sugary foods and drinks. Evidence suggests that a daily intake of two cups of cola is sufficient to elevate the risk of depression [125].

The International Society for Research in Psychiatry recommends the following eating habits to support mental health: 1—adhere to traditional diets such as the Mediterranean, Norwegian, or Japanese diets; 2—increase consumption of FV, whole grains, nuts, and seeds; 3—consume foods rich in Ω3 polyunsaturated fatty acids; 4—replace unhealthy foods with nutritious options; 5—limit intake of processed foods, fast foods, commercial bakery products, and sweets [129]. 

### 3.10. Sedentary Lifestyle

Studies indicate that individuals who are less physically active and engage in minimal exercise have a higher likelihood of developing depression later in life [130,131]. Narrative reviews have highlighted that physical activity can play a preventive role against future depression [113,114]. Meta-analyses of prospective studies have shown that individuals with higher levels of physical activity have a 17% (95% confidence interval (CI), 12%, 21%) lower chance of developing depression compared to those with low activity levels. Another meta-analysis reported a 21% (95% CI, 18%, 25%) reduction in the likelihood of depression associated with higher physical activity [132,133].

Data from the National Health Survey, covering 59,399 people, revealed that a lack of leisure-time physical activity was linked to depression in young males (odds ratio [OR] 1.45; 95% CI, 1.02,2.06), middle-aged individuals (OR 2.38; 95% CI, 1.4, 4.03), and older adults (OR 5.35; 95% CI, 2.14, 13.37) [134]. A similar pattern was observed in older Japanese adults, where lower physical activity levels were associated with a higher incidence of depressive symptoms [135]. In the United States, individuals aged 20 and over who engage only in light physical activity are more likely to experience depression compared to those who participate in vigorous physical activity, with an OR of 3.18 (95% CI, 1.59, 6.37) [136].

Additionally, research conducted across 36 countries found that lower levels of physical activity (defined as less than 150 minutes of moderate to vigorous activity per week) are associated with increased depression rates (OR 1.42; 95% CI, 1.24, 1.63). Studies have also demonstrated that individuals with depression often exhibit lower levels of physical activity and higher levels of sedentary behavior [128,137,138,139]. 

A meta-analysis by Schuch et al. (2018) assessed the impact of physical activity on depression across several prospective cohort studies and found significant protective effects in all age groups: children and adolescents (odds decreased by 10%), adults (odds decreased by 12%), and older adults (odds decreased by 24%). The protective effects varied by region, as follows: Asia (odds decreased by 24%), Europe (odds decreased by 17%), North America (odds decreased by 17%), and Oceania (odds decreased by 35%) [139].

Noetel et al.′s meta-analysis (2024) examined various exercise modalities and their effects on depression. Walking or running were found effective for both men and women, while strength training was more effective for women and yoga for men. Yoga was slightly more beneficial for older adults, whereas strength training was more effective for younger adults [140]. 

### 3.11. Genetic and Epigenetic Factors

Genetic predisposition plays an important role in the development of depression. A family history of depression significantly increases the likelihood of developing the condition. Studies have demonstrated that genetic factors contribute substantially to the risk of depressive disorders [9].

Family and twin studies provide compelling evidence for the role of genetic factors in depression risk. A meta-analysis of twin research data reveals a heritability rate of 37% (95% CI: 31%, 42%), while family studies indicate a two- to threefold increase in the risk of depression among first-degree relatives of depressed patients [141]. Heritability is especially pronounced in severe forms of depression, with severity related to maternal or paternal inheritance of depressive disorders (DD) [142,143,144,145].

The first study aimed at identifying candidate genes related to depressive disorders (DDs) was published in 1978 [146]. Since then, numerous studies worldwide have investigated genes involved in depression progression. Over 100 candidate genes have been analyzed based on neurobiological mechanisms underlying DDs, searching for associations between their alleles and depression risk or symptoms. Despite extensive research, findings have been inconsistent [146].

Advancements in DNA microchip technology have enabled Genome-Wide Association Studies (GWASs), facilitating the search for depression risk factors beyond initial hypotheses. However, even with large sample sizes—including thousands of patients and tens of thousands in meta-analyses—GWASs have not identified specific loci responsible for DD predisposition. These studies have also not clarified the biological mechanisms underlying DD pathogenesis [147,148].

The challenge in pinpointing clear genetic associations and mechanisms indicates that depression is a multifactorial, complex, and heterogeneous disorder. The predisposition to DDs is likely due to the coordinated action of multiple genes, interacting with each other and with environmental factors. Each gene individually contributes relatively little to disease pathogenesis [149].

Additionally, depression development can be influenced by both genetic and epigenetic factors [150]. While genetics pertains to the DNA sequence, epigenetics involves chemical modifications to DNA and associated proteins that affect gene expression without altering the sequence. Epigenetics explains how environmental factors can influence gene expression [151]. Epigenetic mechanisms may mediate enduring increases in depression risk following exposure to adverse life events [150]. These mechanisms are crucial in depression, influencing how genetic and environmental factors interact to increase disease risk [150,151].

**Figure 1:**
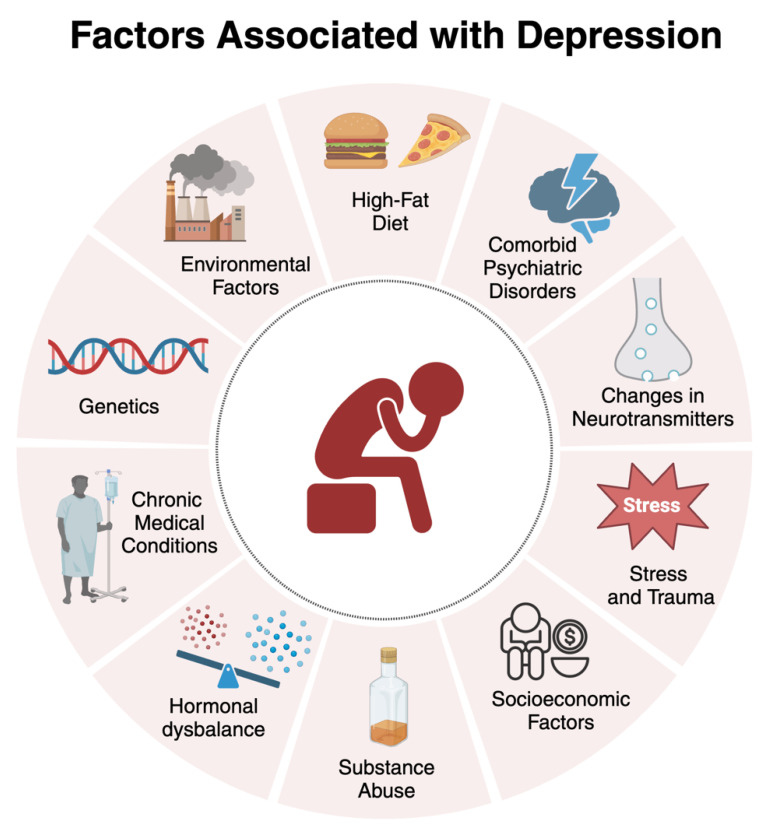
Factors associated with depression. This figure illustrates various factors associated with depression, including genetic and epigenetic factors (inherited traits and gene expression changes), environmental factors (pollution and living conditions), prolonged stress and traumatic experiences, comorbid psychiatric disorders (such as anxiety and bipolar disorder), a high-fat diet, chronic medical conditions (such as hypertension and diabetes), hormonal dysbalance (imbalances in thyroid and sex hormones, cortisol overproduction, etc.), substance abuse, socioeconomic factors (financial stress and low socioeconomic status), and changes in neurotransmitters (alterations in serotonin and dopamine levels).

Sometimes, an individual may not have a genetic predisposition for certain diseases or conditions. However, during gestation, maternal habits can induce epigenetic changes that predispose the individual to such diseases, including depression [152,153]. Although depression has an estimated heritability of 30-40%, indicating that genetics alone does not account for most of the disease risk, environmental factors like childhood adversities and recent stress also play a significant role. Recent studies show that the biological impact of these environmental factors on depression and stress-related disorders is mediated through various epigenetic modifications [153]. Epigenetic mechanisms, such as DNA methylation and histone acetylation, modulate the expression of genes related to inflammation and synaptic plasticity, influencing resilience or susceptibility to depression [154]. Figure 1 illustrates all the aspects discussed so far regarding the factors associated with depression. 

### 3.12. Inflammation

Depression is intricately connected to a systemic immune response, characterized by the activation of inflammatory substances and the migration and regulation of peripheral cells to the central nervous system, facilitated by the permeability of the blood–brain barrier (BBB). Given the multifaceted nature of depression, investigating and monitoring the inflammatory process through the quantification of biomarkers is essential for identifying the disease and its unique characteristics, as well as providing insights into the efficacy of treatments [155,156]. Pre-clinical and clinical research has associated levels of cytokines and antioxidant enzymes with neurotransmitter systems, such as serotonin, norepinephrine, GABA, adrenaline, and cortisol, aiming to develop novel treatment targets due to the resistance often encountered with current therapies. These associations are crucial for enhancing and expanding therapeutic options [155,156]. 

The role of BBB integrity in depression remains unclear: it is not yet determined whether BBB disruption is a primary factor in depression or a consequence of the illness. However, studies have identified dysfunction in the BBB among depressed patients, suggesting that stress and inflammation may play a significant role in its impairment [157,158]. The mechanisms by which stress and inflammation affect BBB integrity are still under investigation, although there is growing evidence of the involvement of cytokines and stress in regulating tight junction proteins that are essential for maintaining BBB integrity, with females being particularly susceptible to these disruptions [157,158].

Increased BBB permeability affects the dynamics of cell migration from the periphery to the brain parenchyma, adapting to the local microenvironment. For instance, microglia can exhibit various behaviors, including pro-inflammatory, anti-inflammatory, and pro-resolving phenotypes, depending on their state. Similarly, mast cells can differentiate in response to the microenvironment, releasing specific mediators that contribute to neuroinflammation and the progression of depression [157,159,160,161]. Astrocytes also play a critical role, providing support to neurons for synaptic functions and helping to prevent neuronal damage and reduced synaptic capacity associated with depressive disorders [158,162]. 

In this context, elevated levels of cytokines, chemokines, interferons, tumor necrosis factors, and growth factors have been linked to mental disorders like depression [155,156]. However, due to variations in study protocols and differing medications among participants, a clear understanding of the relationship between the severity of depression and the modulation by these mediators is still lacking [147,148]. Increased cytokines that initiate the inflammatory cascade, such as interleukin-1 Beta (IL-1β), IL-6, and tumor necrosis factor (TNF), have been associated with severe depression linked to stress [155,156]. Conversely, interleukins like interleukin-10 (IL-10) and interleukin-4 (IL-4) have distinct but interconnected roles in immune system regulation and its relationship with depression [156]. IL-10 is known for its anti-inflammatory and pro-resolving properties, regulating immune responses, and its absence in animal models is associated with depressive behaviors. In humans, reduced IL-10 levels have been observed in those with depressive symptoms, anxiety, and a heightened risk of suicide [156,163,164]. IL-4 influences T cell differentiation and the specialization of microglia toward an anti-inflammatory phenotype [156,165]. Studies suggest that IL-4 is linked to greater resilience against stress-induced depression, promoting increased BDNF and affecting serotonin transporter activity, thereby enhancing serotonin availability in the synaptic cleft [156,166]. Although the exact pathways remain complex and largely unknown, these mediators are emerging as promising therapeutic targets for understanding and treating depression. 

#### 3.12.1. Inflammation and Obesity 

Obesity is characterized by an abnormal or excessive accumulation of fat, which compromises the maintenance of optimal health. The excess of macronutrients in adipose tissues stimulates the release of inflammatory mediators, such as IL-6, while simultaneously reducing the production of adiponectin. This creates a pro-inflammatory state and contributes to oxidative stress [167]. The relationship between obesity and inflammation is intricate and multi-dimensional. Excessive body fat accumulation, particularly in adipose tissue, can lead to metabolic dysfunction and a persistent low-grade inflammatory state [168].

When there is an excess of macronutrients, such as fats and sugars, in the body, adipocytes (fat cells) undergo expansion, which can induce stress within the adipose tissue and potentially lead to hypoxia (lack of oxygen). In response to this stress, adipocytes release inflammatory mediators like TNF-α and IL-6 [169]. These mediators recruit immune cells to the adipose tissue, amplifying the inflammatory response. This phenomenon, known as low-grade inflammation, is characterized by a continuous, relatively mild inflammatory response, contrasting with acute inflammation that occurs in reaction to infection or injury [170].

Obesity also results in reduced production of adiponectin, an anti-inflammatory hormone secreted by adipocytes. Adiponectin possesses anti-inflammatory and insulin-sensitizing properties, and its decrease contributes to insulin resistance and heightened inflammation [170,171]. The inflammation originating in adipose tissue is not confined to this location; the released inflammatory mediators enter systemic circulation, causing a chronic inflammatory state throughout the body. This systemic inflammation can impact various organs and systems, including the liver, heart, and brain [172]. This chronic inflammatory state is linked to several chronic diseases, including cardiovascular disease, type 2 diabetes, insulin resistance, and some forms of cancer. Additionally, inflammation contributes to endothelial dysfunction, which is a precursor to atherosclerosis [173].

The mechanisms by which obesity induces inflammation involve the activation of cellular signaling pathways, such as the NF-κB pathway, which regulates the expression of pro-inflammatory genes. Moreover, the presence of free fatty acids, oxidized lipids, and other metabolic byproducts can activate cellular receptors, triggering inflammatory responses [173].

There exists a bidirectional relationship between depression and increased adiposity; overweight and obesity are associated with a higher prevalence of depression, and conversely, chronic low-grade inflammation, driven by increased adiposity, is a key factor in the pathophysiology of depression [169].

#### 3.12.2. Inflammation and Depression

The relationship between obesity and depression has been explored in various studies, yet the findings remain inconclusive and controversial [174,175]. While some research associates obesity with depression, other research links depression to both low weight and obesity [176,177,178,179]. Additionally, the relationship between these conditions appears to vary across different populations, with some studies identifying associations predominantly in females, while others find stronger connections in males [180,181,182].

A growing body of literature indicates that obesity and depression are intricately connected through a vicious cycle, where each condition exacerbates the other via maladaptive physiological adaptations [183]. Specifically, individuals with overweight or obesity have adipocytes and macrophages in their adipose tissue that produce cytokines and chemokines, which can cross the blood–brain barrier and induce neuroinflammation [184]. This obesity-induced neuroinflammation impacts multiple brain regions, including the hippocampus, cortex, brainstem, and amygdala. Research suggests that the heightened secretion of pro-inflammatory cytokines disrupts metabolic processes, neurotransmitter function, and brain plasticity [185]. Furthermore, long-term obesity, particularly when initiated in prepuberty and maintained into adulthood through a high-fat diet, is associated with increased inflammation in the prefrontal cortex, a factor potentially linked to depression.

Obesity is also correlated with a higher incidence of central nervous system disorders, such as depression, and a decline in cognitive abilities [186]. Studies have reported that neuroinflammation is closely linked to cognitive impairment, with middle-aged obese adults being at a higher risk of cognitive decline [187], particularly in areas of executive function and memory [188].

Adipose tissue, far from being merely a passive storage site for energy, is now recognized as a dynamic organ that plays a critical role in various vital physiological processes. It comprises diverse cell types, including adipocytes, pericytes, pre-adipocytes, vascular endothelial cells, macrophages, and fibroblasts. Adipose tissue is involved in energy storage, adipokine production, and the regulation of energy balance, in addition to playing a significant role in the endocrine system [187]. While adipokines have been shown to act as effective modulators in depressive states and anxiety, their precise mechanism in modulating depressive behavior remains to be fully elucidated [188]. Additionally, interventions targeting oxidative stress, as demonstrated in models of Alzheimer’s disease, have shown potential benefits in cognitive function and memory, highlighting the importance of addressing neuroinflammation in obesity-related depression [189,190].

Leptin and adiponectin, two hormones associated with obesity, play crucial roles in brain function and are linked to both obesity and depression. Leptin, primarily produced by adipose tissue, regulates eating behavior and body weight, and is associated not only with obesity but also with depression [191]. Research has demonstrated that leptin levels, as well as body mass index, are significantly higher in patients with moderate to severe depression compared to those with mild or no depression. Even after adjusting for age, sex, and ethnicity, leptin levels remain a strong predictor of depression [192].

In contrast, adiponectin levels are reduced in both adipose tissue and blood of obese individuals, although the expression of adiponectin receptors is increased [193]. A study by Liu and colleagues [194] found that in a chronic social defeat stress model of depression, plasma adiponectin levels decreased, which led to a reduction in social interaction duration. The study also suggested that lower adiponectin levels may increase susceptibility to social aversion, anhedonia, and learned helplessness, as well as negatively impact the HPA axis via impaired glucocorticoid-mediated feedback [195]. 

The gut microbiota has also emerged as a significant player in the obesity–depression relationship, with changes in gut microbiota composition potentially influencing the function of the HPA axis and contributing to hormonal dysregulation. This interaction between the HPA axis and gut microbiota plays a critical role in various mental and gastrointestinal conditions. Current hypotheses suggest that the gut microbiota may play a role in the pathogenesis of obesity-associated depression [195,196]. Evidence points to a significant interaction between gut microbiota composition, obesity, and systemic inflammation [197,198]. The gut–brain axis is increasingly recognized as a bidirectional neuro-humoral communication system, essential for integrating brain and gastrointestinal functions. Moreover, metabolites produced by gut microbiota have been implicated in behavioral modulation and are associated with depression [197,198].

In this context, diets high in fat, chronic inflammation, and neuronal responses are factors that influence microbiota quality and contribute to the development and worsening of obesity and depression. However, the use of specific prebiotics and probiotics appears to help regulate these conditions [198].

## 4. Obesity and Depression

Chronic low-grade inflammation, characterized by the secretion of pro-inflammatory cytokines such as TNF-α and IL-6, is a central link between obesity and depression, establishing a vicious cycle where each condition can exacerbate the other [199]. In obese individuals, the excess adipose tissue contributes to this persistent inflammatory state. Cytokines released by adipose tissue cells, including adipocytes and macrophages, have the ability to cross the BBB, inducing neuroinflammation, a phenomenon often associated with depression [200].

The integrity of the BBB is essential for protecting the central nervous system from harmful external influences. However, in obesity, increased BBB permeability facilitates the infiltration of inflammatory cells and mediators into the brain, exacerbating local inflammatory processes. This dysfunction can impair neuronal function and promote behavioral changes typical of depressive disorders. Although not yet fully understood, BBB dysfunction in depressed individuals appears to be linked to factors such as stress and systemic inflammation [201,202]. This ongoing inflammatory state interferes with key neurotransmitters involved in mood regulation, such as serotonin and dopamine, thereby contributing to the emergence of depressive symptoms. This disruption in neurotransmitter balance may partially explain the high prevalence of depressive disorders among obese individuals [203,204].

Adipokines, hormones produced by adipose tissue, play significant roles at the intersection of obesity and depression. Leptin, for example, is crucial in regulating feeding behavior and is involved in inflammatory signaling within the brain. Elevated leptin levels have been associated with greater severity of depression. In contrast, adiponectin, which has anti-inflammatory properties, is reduced in obese individuals, potentially exacerbating inflammation and increasing susceptibility to depression [205,206,207,208]. Beyond mood, inflammation also impacts cognitive function. Neuroinflammation, often promoted by obesity, has been linked to declines in cognitive functions, particularly in memory and executive functions [75]. This impact is especially relevant in middle-aged individuals, where obesity may lead to an increased risk of early cognitive decline [209].

**Figure 2:**
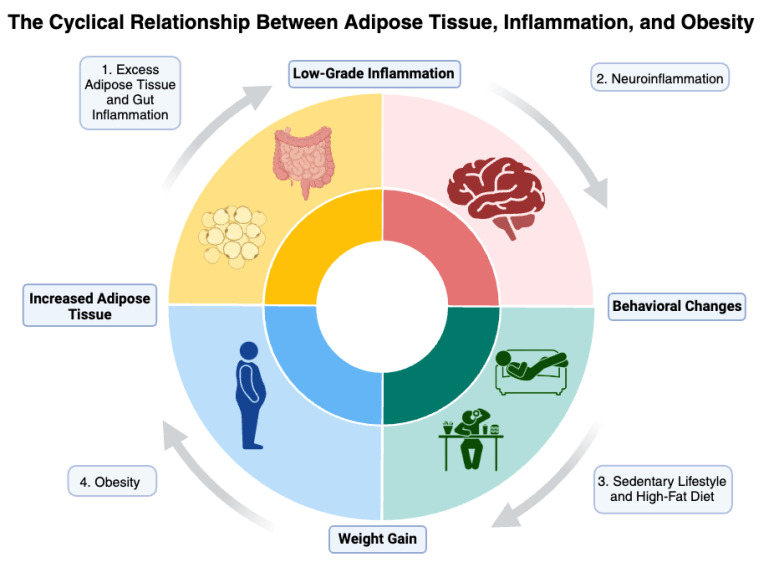
The cyclical relationship between adipose tissue, inflammation, and obesity. This figure depicts the cyclical relationship between excess adipose tissue and gut inflammation (yellow), neuroinflammation (red), sedentary lifestyle and high-fat diet (green), and obesity (blue). Excess adipose tissue and gut inflammation contribute to systemic low-grade inflammation, which leads to neuroinflammation. Neuroinflammation then results in behavioral changes, promoting a sedentary lifestyle and high-fat diet, leading to weight gain and obesity. Obesity further increases adipose tissue, perpetuating the cycle.

Recently, the gut microbiota has been recognized as a key factor in the link between obesity, inflammation, and depression [210]. Changes in the composition of the microbiota can influence systemic inflammation and the function of the HPA axis, contributing to hormonal dysfunctions and increased susceptibility to depressive disorders. The regulation of the microbiota through specific diets and the use of prebiotics and probiotics emerge as promising strategies to mitigate the effects of obesity on mental health [210,211]. Figure 2 illustrates the complex relationship between obesity and depression. 

## 5. Conclusions

The relationship between obesity and depression is influenced by various factors, including inflammation, neurochemical changes, and social challenges like weight stigma. Obesity increases the risk of depression, and depression can, in turn, worsen obesity through appetite issues and reduced activity. Additionally, exposure to pesticides may contribute to this link by disrupting endocrine and neurological functions. Understanding these connections is essential for creating effective treatments that address both conditions. Further research should explore these links to develop better therapeutic strategies.

## Data Availability

Data sharing is not applicable to this article as no new data were created or analyzed in this study.

## Abbreviations

ACEs: Adverse Childhood Experiences; AR: Adverse Reaction; BBBD: Blood-Brain Barrier Dysfunction; BMI: Body Mass Index; CNS: Central Nervous System; DC: Depression Condition; DSM: Diagnostic and Statistical Manual; GAD: Generalized Anxiety Disorder; HPA: Hypothalamic–Pituitary–Adrenal; IL: Interleukin; MDD: Major Depressive Disorder; NP: Neuropsychiatric; PM: Preventive Measures; QoL: Quality of Life; RCT: Randomized Controlled Trial; SD: Standard Deviation; SES: Socioeconomic Status; SUD: Substance Use Disorder; US: United States.
